# Risk of Major Adverse Cardiovascular Events With Dolutegravir Versus Efavirenz-Based Antiretroviral Therapy: Emulated Target Trials Using Routine, De-identified Data From South Africa

**DOI:** 10.1093/cid/ciag155

**Published:** 2026-03-04

**Authors:** Jienchi Dorward, Xolani Masombuka, Lara Lewis, Claudia Pastellides, Johan van der Molen, Kwabena Asare, Kwena Tlhaku, Jennifer Anne Brown, Christian Bottomley, Dave Jacobs, Shirley Collie, Nigel Garrett

**Affiliations:** Nuffield Department of Primary Care Health Sciences, University of Oxford, Oxford, Oxfordshire, United Kingdom; Centre for the AIDS Programme of Research in South Africa (CAPRISA), University of KwaZulu-Natal, Durban, KwaZulu-Natal, South Africa; Health Intelligence Unit, Discovery Health, Johannesburg, Gauteng, South Africa; Centre for the AIDS Programme of Research in South Africa (CAPRISA), University of KwaZulu-Natal, Durban, KwaZulu-Natal, South Africa; Health Intelligence Unit, Discovery Health, Johannesburg, Gauteng, South Africa; Centre for the AIDS Programme of Research in South Africa (CAPRISA), University of KwaZulu-Natal, Durban, KwaZulu-Natal, South Africa; Centre for the AIDS Programme of Research in South Africa (CAPRISA), University of KwaZulu-Natal, Durban, KwaZulu-Natal, South Africa; Department of Infectious Disease Epidemiology and International Health, London School of Hygiene & Tropical Medicine, London, United Kingdom; Centre for the AIDS Programme of Research in South Africa (CAPRISA), University of KwaZulu-Natal, Durban, KwaZulu-Natal, South Africa; Discipline of Public Health Medicine, School of Nursing and Public Health, University of KwaZulu-Natal, Durban, KwaZulu-Natal, South Africa; Nuffield Department of Primary Care Health Sciences, University of Oxford, Oxford, Oxfordshire, United Kingdom; Centre for the AIDS Programme of Research in South Africa (CAPRISA), University of KwaZulu-Natal, Durban, KwaZulu-Natal, South Africa; Department of Infectious Disease Epidemiology and International Health, London School of Hygiene & Tropical Medicine, London, United Kingdom; Health Intelligence Unit, Discovery Health, Johannesburg, Gauteng, South Africa; Health Intelligence Unit, Discovery Health, Johannesburg, Gauteng, South Africa; Centre for the AIDS Programme of Research in South Africa (CAPRISA), University of KwaZulu-Natal, Durban, KwaZulu-Natal, South Africa; Discipline of Public Health Medicine, School of Nursing and Public Health, University of KwaZulu-Natal, Durban, KwaZulu-Natal, South Africa; Desmond Tutu HIV Centre, University of Cape Town, Cape Town, South Africa

**Keywords:** HIV, dolutegravir, antiretroviral therapy, cardiovascular disease, South Africa

## Abstract

**Background:**

Integrase inhibitors, including dolutegravir, may increase risk of major adverse cardiovascular events (MACEs). However, limited data exists from low- and middle-income countries, where tenofovir disoproxil fumarate, lamivudine, and dolutegravir (TLD) has largely replaced tenofovir disoproxil fumarate, emtricitabine, and efavirenz (TEE).

**Methods:**

We used de-identified data from a South African managed healthcare organization from people with HIV (PWH) without cardiovascular disease, who either initiated TEE or TLD between April 2020 and Dec 2023 (initiation cohort) or were receiving TEE in April 2020 and eligible for TLD (transition cohort). In the initiation cohort, we emulated a target trial using pooled logistic regression models with inverse probability of treatment weights and bootstrapped CIs to compare estimated 3-year MACE risk between TLD versus TEE. In the transition cohort, we used similar methods in 44 emulated monthly sequential trials, comparing MACE risk in people transitioned to TLD with those remaining on TEE.

**Results:**

In the initiation cohort, 7310 PWH initiated TLD (n = 3711) or TEE (n = 3599). Median follow-up was 21 months (IQR 10–33), with 18 MACEs with TLD (3-year risk 0.78%, 95% CI .38–1.11) and 28 with TEE (3-year risk 1.03%, 0.63–1.55; RR 0.75, 0.31–1.30; RD −0.25, −0.94–0.24). In the transition cohort, 22 338 people contributed to 2837 person-trials with TLD and 706 615 with TEE. Median follow-up was 25 months (14–36), with 19 MACEs with TLD (3-year risk 0.97%, 0.52–1.62) and 5420 with TEE (3-year risk 1.17%, 0.99–1.37; RR 0.83, 0.45–1.39; RD −0.20, −0.66–0.44).

**Conclusions:**

Among PWH in South Africa, we found no increased MACE with TLD.

## Teaser

Dolutegravir may increase the risk of major adverse cardiovascular events (MACE). We emulated target trials using de-identified healthcare data from 29 648 people living with HIV in South Africa and found no increased risk of MACE with dolutegravir.

The integrase strand transferase inhibitor (INSTI) dolutegravir is recommended for first- and second-line antiretroviral therapy (ART) in over 118 low- and middle-income countries (LMICs) and is used by over 20 million people with HIV (PWH) [1, 2]. Dolutegravir has better efficacy, fewer side effects, and a higher genetic barrier to resistance, compared to the previously recommended efavirenz [3]. However, there are concerns regarding a potential association between INSTI use and major adverse cardiovascular events (MACEs), with an observational study in European and Australian cohorts finding increased MACE risk in the first 24 months of INSTI use [4]. Furthermore, several African clinical trials found that dolutegravir was associated with greater weight gain than efavirenz, particularly among women [5, 6], although whether this translates into increased MACE risk remains unclear. Findings from observational studies, which have tended to focus on INSTIs as a group rather than dolutegravir alone, have been mixed [4, 7–10] and conducted predominantly in European or North American populations. In African populations, which contain the largest number of people taking dolutegravir and higher proportions of women, studies have not been sufficiently powered to evaluate MACEs [11, 12].

We hypothesized a priori that dolutegravir may increase cardiovascular risk and so aimed to assess whether dolutegravir increases MACE risk compared to the previously recommended efavirenz among PWH in South Africa.

## METHODS

We used observational data to emulate target trials, a methodology that aims to reduce bias when using observational data for causal inference [13, 14]. Following reporting recommendations [15], we specify key components of the hypothetical target trials that we aimed to emulate, before describing the observational data and emulation methods.

### Target Trial Specifications

We emulated target trials in 2 cohorts, people initiating ART (initiation cohort) and people already receiving first-line ART (transition cohort) (Table 1). For the initiation target trial, eligible participants would be PWH aged ≥18 years, without known cardiovascular disease (CVD), and newly initiating ART, and would be randomized at baseline to initiate open-label tenofovir disoproxil fumarate, lamivudine, and dolutegravir (TLD) or tenofovir disoproxil fumarate, emtricitabine, and efavirenz (TEE) (Table 1). For the transition target trial, eligible participants would be PWH aged ≥18 years already receiving TEE, without current viremia >1000 copies/mL, without known CVD, and eligible for transition to first-line TLD. People would be randomized at baseline to either continue TEE, or be transitioned to TLD. In both trials, the primary outcome, MACE (cardiovascular death or hospitalization), would be assessed up to 36 months, with censoring at ART gap >6 months, death, withdrawal, or study end. The primary analysis would be an intention-to-treat analysis, with a secondary per-protocol analysis. Because the effect of dolutegravir on weight gain is greater among women [5, 6] and the excess risk of MACE due to HIV may be greater among women [16], we planned a sensitivity analysis to examine sub-group effects by gender on the risk of MACE with TLD. The 3-year estimated cumulative risk of MACE in each arm would be estimated using hazards estimated using pooled logistic regression models and compared using risk ratios and risk differences, with 95% CIs calculated using 500 bootstrap samples. To reduce bias from informative censoring, the pooled logistic regression model would be weighted for the inverse probability of censoring, estimated using baseline and time varying covariates. In the per-protocol analysis, participants would additionally be censored if they change ART, with additional weighting for censoring due to ART change.

**Table 1.** Specification of the Target Trials

	Initiation Cohort Analysis	Transition Cohort Analysis
Eligibility criteria	Person with HIV aged ≥18 y old, without previous known cardiovascular disease, and newly initiating ART	Person with HIV aged ≥18 y old, already receiving TEE, without current viremia (>1000 copies/mL), without previous known cardiovascular disease, and eligible for transition to first-line TLD
Treatment strategies	Initiated on TLD or TEE	Transition immediately to TLD or continue TEE
Treatment assignment	Randomized, with clinician and participant aware of allocation	
Outcome	Major adverse cardiovascular events (MACE) consisting of cardiovascular death or hospitalization	
Follow-up	Followed up until the earliest of treatment interruption, death, withdrawal, MACE, or 36 m	
Causal contrasts of interest	Primary analysis would be an intention to treat analysis, with a secondary per-protocol analysis	
Analysis plan	Pooled logistic regression model to estimate time-varying hazards, which are used to calculate the estimated 3-y risk of MACE under TLD versus TEE, compared with risk ratios and risk differences	Pooled logistic regression model, to estimate time-varying hazards, which are used to calculate the estimated 3-y risk of MACE under TLD versus TEE, compared with risk ratios and risk differences

### Observational Data Source and Data Management

We used de-identified, routinely collected data from a South African managed healthcare organization (Discovery Health, Johannesburg, South Africa) that collects and securely processes healthcare data on members for the purposes of administering medical aid schemes and funding healthcare service provision. In this scheme, ART is provided through private general practitioners or infectious diseases specialists, normally following Southern African HIV Clinician Society or South African National Department of Health guidelines [17, 18]. Dolutegravir started to be widely used for first-line ART from early 2020, when it was introduced into the public sector in a fixed dose combination pill of TLD, replacing a fixed dose combination pill of TEE. Initially, TLD use was restricted in women of child-bearing potential due to safety concerns, but in July 2021, this recommendation was lifted, and TLD became the preferred first-line regimen for all PWH [19]. Viral load testing was recommended 6 monthly or annually, and transition to first-line dolutegravir was only recommended if people had a suppressed viral load of <50 copies/mL in the previous 6 months, or consecutive viral loads between 50 and 999 copies/mL [17]. After July 2022, these viral suppression criteria were removed [20].

For all members, the managed healthcare organization collects and processes data on self-reported medical conditions at enrollment, new diagnoses during follow-up, claims for medication prescriptions (including ART), hospitalization diagnostic codes, laboratory investigations and results, and cause of death. Data on confirmed chronic conditions, verified through physician documentation and valid claims containing appropriate diagnosis codes, was collected, cleaned, cross-checked between different databases, anonymized, and securely processed by the managed healthcare organization prior to extraction for analysis.

### Participants

For the initiation cohort, we included PWH aged ≥18 years, without known CVD, and newly initiating TLD or TEE first-line ART within the managed healthcare cohort, between 1 April 2020 and 31 December 2023. This allowed at least 6 months of follow-up until 30 June 2024, with 3 months of data capture “run-off” before the data cut on 30 September 2024. We excluded people with known previous ART exposure, <6 months of insurance scheme membership (as it was not possible to know if they joined the scheme while already receiving ART) or suppressed viral load <1000 copies/mL at initiation, which may suggest current or recent ART exposure. For the transition cohort, we included PWH aged ≥18 years, without known CVD, and already receiving TEE in the managed healthcare cohort in April 2020, and followed them until 30 June 2024, again to allow 3 months of data capture “run-off” before the data cut.

### Variables

#### Outcomes

We used cause of death and hospital admission codes to define the primary endpoint of MACE as a composite of death related to acute myocardial infarction or stroke, or hospital admission for acute myocardial infarction, unstable angina, stroke (ischemic, hemorrhagic, or undetermined), transient ischemic attack, peripheral arterial ischemia and coronary, carotid or peripheral artery revascularization (eg angioplasty, stenting, coronary bypass surgery, carotid endarterectomy). People who withdrew from the medical scheme were defined as lost to follow-up on the date of withdrawal.

#### Exposure Variable

We used ART claims data to determine ART exposure at baseline and during follow-up. We used the first TLD or TEE claim to determine the date of initiation (initiation cohort) and the most recent claim in April 2020 to determine baseline ART exposure in the transition cohort. We censored follow-up of anyone with a gap in ART claims >6 months due to uncertainty in ART exposure and continued scheme activity regarding claims for hospitalizations.

#### Covariates

At baseline and throughout follow-up, we used laboratory data to determine CD4 T-cell counts and viral loads and chronic illness benefit application forms and disease specific claims to determine CVD, hypercholesterolemia, hypertension, diabetes mellitus, pregnancy, and tuberculosis episodes. We determined statin use using prescription claims and used scheme benefit level as a proxy for socioeconomic status. Where data was missing, we included a category for missing data. We did not include smoking status as data was missing for >90% of participants.

### Statistical Analysis

In the initiation cohort intention-to-treat analysis, we emulated randomization between TLD or TEE using stabilized inverse probability of treatment weights (IPTWs), calculated using propensity scores from a logistic regression model with treatment assignment as the outcome and potential confounders at baseline as covariates [21] (age, gender, province, scheme benefit level, initiation period [in quarterly intervals], CD4 count, viral load, known TB, known pregnancy, known diabetes, known hypertension, known hypercholesterolemia, statin use). We accounted for informative censoring with stabilized inverse probability of censoring weights (IPCWs), calculated using a pooled logistic regression model for the monthly risk of censoring, with baseline (ART regimen and the same variables as the IPTW model) and time-updated variables (follow-up time in months, viral load, CD4, statin use, pregnancy status, and incident tuberculosis, diabetes mellitus, hypercholesterolemia, and hypertension) as covariates. We then estimated the hazards of MACE by fitting a weighted pooled logistic regression model with a time-varying intercept, a treatment assignment variable and a treatment–time interaction. Weights were calculated by multiplying the IPTWs and IPCWs. We used the model to predict monthly outcomes under the scenarios of all participants receiving TEE, and all participants receiving TLD, and calculated the estimated 3-year risk of MACE with TEE and TLD, the risk ratio and risk difference, with 95% CIs estimated from 500 bootstrap samples. For the per-protocol analysis, we used a similar approach, but the final weights included an additional set of stabilized IPCWs for the monthly risk of censoring due to change in ART regimen, using the same baseline and time-updated variables as covariates. We truncated all weights at the 99th percentile.

For the transition cohort intention-to-treat analysis, we emulated 44 sequential target trials [22], each using a different baseline month from May 2020 to December 2023. We emulated randomization, between remaining on TEE versus transitioning in the baseline month to TLD, using IPTW, calculated using covariate values from baseline of the respective trial (age, gender, province, scheme benefit level, baseline CD4 count, baseline viral load, TB status, pregnancy status, known diabetes, known hypertension, known hypercholesterolemia, statin use). As per the target trial eligibility criteria, people who were transitioned to TLD were excluded from subsequent trials, but people who remained on TEE could have been included in subsequent trials, meaning individuals could appear in multiple trials. We then used similar methods to the initiation cohort analysis (combining IPTWs and IPCWs and with an additional time term for trial included in the outcome model) to estimate the 3-year risk of MACE under TLD and TEE, and the risk difference and risk ratio, with 500 bootstrap samples to estimate 95% CIs. For the per-protocol analysis, individuals within each trial were additionally censored upon ART regimen changes, and the outcome model was again weighted for IPTWs multiplied by IPCWs and IPCWs for censoring due to ART regimen change.

In both the initiation and transition analyses, we conducted sensitivity analyses including body mass index (BMI) (with a category for missing) in the IPTW (and IPCW) models and further sensitivity analyses with an interaction term between treatment assignment and gender in the outcome model. We did not conduct power calculations before starting these analyses.

We analyzed data using R 4.4.0 (R Foundation for Statistical Computing, Vienna, Austria), with code available in the Supplementary Appendix.

## RESULTS

### Initiation Cohort

**Figure 1.**
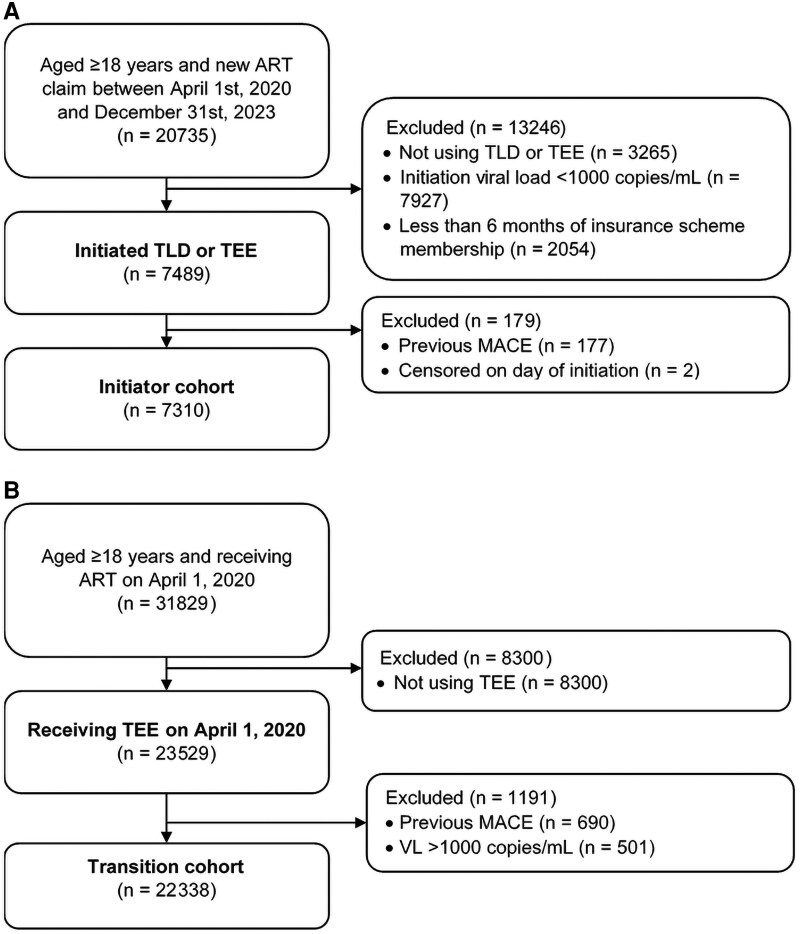
Flow diagram of A) initiation and B) transition cohorts

In the initiation cohort, between 1 April 2020 and 31 December 2023, 7310 people initiated TLD (n = 3711) and TEE (n = 3599) (Figure 1). Median (IQR) age was 38 (32–44) years, 57.0% were female, and 14.2% had a recorded CVD risk factor (Table 2). Baseline BMI was available for 1993 (27.3%) of participants; of these, 1394 (70.0%) were overweight, obese, or severely obese. The TLD group had fewer women (54.4% vs 59.8%), pregnant people (5.3% vs 10.8%), and people with missing baseline BMI (66.3% vs 79.4%) versus TEE. However, among those with BMI recorded, distributions were similar. There was a higher proportion of people who were initiated later in the study period (eg Oct–Dec 2023 8.8% vs 2.3%) in the TLD versus TEE groups. After IPTW, baseline covariates were well balanced between the 2 groups (Table 2).

**Table 2.** Baseline Characteristics of Initiation Cohort and Emulated Target Trial Pseudo-population After Inverse Probability of Treatment Weighting

		Initiation Cohort			Target Trial Pseudo-population		
Variable	Level	TEE (n = 3599, 49.2%)	TLD (n = 3711, 50.8%)	Total (n = 7310)	TEE (n = 3600)	TLD (n = 3674)	Standardized Mean Difference
Age, years	Mean (SD)	38.7 (8.9)	39.0 (8.9)	38.8 (8.9)	38.92 (8.87)	39.02 (9.01)	0.011
Gender	Female (%)	2151 (59.8)	2017 (54.4)	4168 (57.0)	2038.4 (56.6)	2064.2 (56.2)	0.009
Province	Gauteng	1502 (41.7)	1665 (44.9)	3167 (43.3)	1544.5 (42.9)	1587.5 (43.2)	0.009
	Eastern Cape	223 (6.2)	232 (6.3)	455 (6.2)	219.3 (6.1)	221.3 (6.0)	…
	KwaZulu-Natal	793 (22.0)	697 (18.8)	1490 (20.4)	741.1 (20.6)	747.0 (20.3)	…
	Mpumalanga	277 (7.7)	284 (7.7)	561 (7.7)	287.2 (8.0)	294.1 (8.0)	…
	Other	558 (15.5)	537 (14.5)	1095 (15.0)	535.7 (14.9)	551.0 (15.0)	…
	Western Cape	246 (6.8)	296 (8.0)	542 (7.4)	271.9 (7.6)	273.7 (7.4)	…
Initiation time period	Apr–Jun 2020	338 (9.4)	118 (3.2)	456 (6.2)	225.2 (6.3)	229.2 (6.2)	0.019
	Jul–Sep 2020	244 (6.8)	104 (2.8)	348 (4.8)	169.6 (4.7)	165.6 (4.5)	…
	Oct–Dec 2020	277 (7.7)	115 (3.1)	392 (5.4)	191.8 (5.3)	189.2 (5.1)	…
	Jan–Mar 2021	653 (18.1)	267 (7.2)	920 (12.6)	452.0 (12.6)	457.5 (12.4)	…
	Apr–Jun 2021	286 (7.9)	190 (5.1)	476 (6.5)	233.3 (6.5)	237.5 (6.5)	…
	Jul–Sep 2021	266 (7.4)	189 (5.1)	455 (6.2)	223.2 (6.2)	226.8 (6.2)	…
	Oct–Dec 2021	222 (6.2)	161 (4.3)	383 (5.2)	187.8 (5.2)	194.3 (5.3)	…
	Jan–Mar 2022	256 (7.1)	252 (6.8)	508 (6.9)	250.4 (7.0)	256.6 (7.0)	…
	Apr–Jun 2022	186 (5.2)	239 (6.4)	425 (5.8)	207.2 (5.8)	214.0 (5.8)	…
	Jul–Sep 2022	171 (4.8)	312 (8.4)	483 (6.6)	240.5 (6.7)	246.3 (6.7)	…
	Oct–Dec 2022	184 (5.1)	348 (9.4)	532 (7.3)	270.0 (7.5)	273.6 (7.4)	…
	Jan–Mar 2023	182 (5.1)	379 (10.2)	561 (7.7)	277.7 (7.7)	284.5 (7.7)	…
	Apr–Jun 2023	133 (3.7)	327 (8.8)	460 (6.3)	227.0 (6.3)	234.3 (6.4)	…
	Jul–Sep 2023	119 (3.3)	384 (10.3)	503 (6.9)	251.2 (7.0)	257.5 (7.0)	…
	Oct–Dec 2023	82 (2.3)	326 (8.8)	408 (5.6)	192.7 (5.4)	207.5 (5.6)	…
Scheme benefit level	Network/PMB	1486 (41.3)	1454 (39.2)	2940 (40.2)	1405.4 (39.0)	1439.2 (39.2)	0.007
	High day to day	157 (4.4)	193 (5.2)	350 (4.8)	186.3 (5.2)	184.4 (5.0)	…
	Low day to day	1956 (54.3)	2064 (55.6)	4020 (55.0)	2007.8 (55.8)	2050.9 (55.8)	…
Initiation VL, copies/mL	1000–9999	420 (11.7)	362 (9.8)	782 (10.7)	381.5 (10.6)	393.1 (10.7)	0.013
	10 000–999 999	1539 (42.8)	1680 (45.3)	3219 (44.0)	1583.9 (44.0)	1622.3 (44.1)	…
	>999 999	156 (4.3)	279 (7.5)	435 (6.0)	209.3 (5.8)	222.2 (6.0)	…
	Missing	1484 (41.2)	1390 (37.5)	2874 (39.3)	1424.8 (39.6)	1436.9 (39.1)	…
Initiation CD4 count, cells/µL	0–49	203 (5.6)	306 (8.2)	509 (7.0)	252.2 (7.0)	259.2 (7.1)	0.012
	50–199	378 (10.5)	481 (13.0)	859 (11.8)	412.8 (11.5)	431.9 (11.8)	…
	200–349	381 (10.6)	387 (10.4)	768 (10.5)	382.8 (10.6)	382.3 (10.4)	…
	350–499	298 (8.3)	311 (8.4)	609 (8.3)	295.1 (8.2)	300.6 (8.2)	…
	≥500	387 (10.8)	418 (11.3)	805 (11.0)	401.0 (11.1)	413.7 (11.3)	…
	Unknown	1952 (54.2)	1808 (48.7)	3760 (51.4)	1855.6 (51.6)	1886.9 (51.4)	…
TB at ART initiation	Yes (%)	84 (2.3)	114 (3.1)	198 (2.7)	101.7 (2.8)	101.7 (2.8)	0.003
Pregnant at ART initiation	Yes (%)	390 (10.8)	198 (5.3)	588 (8.0)	281.8 (7.8)	259.0 (7.0)	0.030
Hypercholesterolemia	Yes (%)	188 (5.2)	216 (5.8)	404 (5.5)	201.4 (5.6)	206.7 (5.6)	0.001
Hypertension	Yes (%)	352 (9.8)	374 (10.1)	726 (9.9)	361.3 (10.0)	367.5 (10.0)	0.001
Diabetes mellitus	Yes (%)	127 (3.5)	96 (2.6)	223 (3.1)	111.7 (3.1)	111.0 (3.0)	0.005
Chronic kidney disease	Yes (%)	2 (0.1)	3 (0.1)	5 (0.1)	2.1 (0.1)	2.7 (0.1)	0.006
CVD risk factor^a,b^	Yes (%)	500 (13.9)	535 (14.4)	1035 (14.2)	…	…	…
Statin use	Yes (%)	81 (2.3)	93 (2.5)	174 (2.4)	86.1 (2.4)	88.6 (2.4)	0.001
Initiation BMI (including missing)^b^	Missing	2856 (79.4)	2461 (66.3)	5317 (72.7)	2704.3 (75.1)	2577.5 (70.1)	0.116
	Underweight/normal	225 (6.3)	374 (10.1)	599 (8.2)	276.1 (7.7)	325.5 (8.9)	…
	Overweight	244 (6.8)	446 (12.0)	690 (9.4)	294.0 (8.2)	388.6 (10.6)	…
	Obese/severely obese	274 (7.6)	430 (11.6)	704 (9.6)	325.1 (9.0)	383.0 (10.4)	…

^a^Composite of hypercholesterolemia, hypertension, diabetes, and chronic kidney disease.

^b^Not included in model to calculate IPTWs in main analysis.

People were followed up for a median of 21 months (IQR 10–33) until censoring, for a total of 12 467 person-years. During follow-up, 196 (5.3%) people who were initiated on TLD were changed to another dolutegravir-based regimen (n = 84), or an efavirenz-based regimen (n = 59) or another non-dolutegravir-based regimen (n = 53), after a median of 212 days (IQR 90–426) from ART initiation. 636 (17.7%) people initiated on TEE were changed to TLD (n = 536), another dolutegravir-based regimen (n = 28), another efavirenz-based regimen (n = 8) or another non-efavirenz-based regimen (n = 64), after a median of 306 days (IQR 125–634).

By the end of follow-up 4618 (63.2%) remained in care, 1765 (24.1%) had withdrawn, 739 (10.1%) experienced a gap in ART >6 months, 142 (1.9%) had died, and 46 (0.6%) had experienced a MACE. MACEs consisted of stroke (n = 22), unstable angina (n = 12), coronary revascularization (n = 8), and acute myocardial infarction (n = 4). There were 18 MACEs with TLD after a median of 8 months (IQR 5–17), and 28 with TEE after a median of 4 months (IQR 3–13). The cumulative incidence of MACE at 3-years was 0.91% (95% CI .40–1.42) with TLD and 1.00% (0.61–1.39) with TEE.

#### Initiation Cohort Emulated Target Trial

In the emulated target trial intention to treat analysis, the estimated 3-year risk of MACE was 0.78% (0.38–1.11) with TLD and 1.03% (0.63–1.55) with TEE (risk ratio [RR] 0.75, 95% CI .31–1.30; risk difference [RD] −0.25 (−0.94 to 0.24), (Table 3). In the per-protocol analysis, the estimated 3-year risk of MACE was 0.66% (0.30–1.17) with TLD and 1.00% (0.60–1.45) with TEE (RR 0.66 [0.27–1.52]; RD −0.34% [−0.93 to 0.36]). In a sensitivity analysis including baseline BMI (with a category for missing) in the model to calculate IPTWs, there was no meaningful change in results (intention-to-treat analysis RR 0.75 [0.30–1.26], RD −0.25% [−0.98 to 0.23]). In a sensitivity analysis with an interaction term between gender and treatment allocation in the MACE outcome model, there was no evidence of a difference in the effect of TLD on MACE in women (RR 0.70, 0.24–2.03) and in men (RR 0.80, 0.27–1.99; Supplementary Figure 1*A*).

### Transition Cohort

In the transition cohort, we included 22 338 individuals who were receiving TEE in April 2020 and were potentially eligible for transition to TLD (Figure 1). Median (IQR) age was 41 (36–47) years and 61.7% were female (Supplementary Table 1). People were followed up for a median of 51 (25–51) months until censoring or database closure, for a total of 72 514 person-years. By 30 June 2024, 343 (1.5%) had died, 2546 (11.4%) had an ART gap >6 months, 6700 (30.0%) had withdrawn, 12 494 (55.9%) remained in care without experiencing a MACE, and 255 (1.1%) had experienced a MACE. MACEs occurred after a median of 26 months (IQR 13–38) and included hospitalization from stroke (n = 109, 42.7%), unstable angina (n = 102, 40.0%), acute myocardial infarction (n = 24, 9.4%), coronary revascularization (n = 15, 5.9%) and peripheral arterial ischemia (n = 3, 1.2%), and death from acute myocardial infarction (n = 1, 0.4%) and stroke (n = 1, 0.4%). During follow-up, 2837 were transitioned to TLD while not viremic after a median of 30 (IQR 15–39) months and were included in the TLD arms of the emulated monthly sequential target trials. Those who were not viremic and who remained on TEE at the same time point could be included in subsequent sequential trials, meaning individuals appeared in multiple trials.

#### Transition Cohort Emulated Target Trial

We therefore now describe the sequential trials' population using person-trials. There were 2837 person-trials in the TLD arms and 706 615 in the TEE arms of the 44 trials. The proportion of women (57.9% vs 62.2%), person-trials with CD4 count ≥500 cells/µL (53.7% vs 59.0%), baseline cardiovascular risk factors (12.3% vs 15.9%), and missing BMI (66.1% vs 76.4%) were lower with TLD versus TEE (Table 3). However, distributions were similar among those with recorded BMI. Person-trials were slightly older (44 vs 43 years) and had been on ART for longer (7 vs 6 years) with TLD versus TEE. After IPTW, baseline characteristics were similar between groups (Table 4).

**Table 3.** Observed and Estimated 3-Year Risk of Major Adverse Cardiovascular Events With TLD Versus TEE in the Initiation and Transition Cohort Emulated Target Trials

	N	Median FU Time, Years (IQR)	Person-Years FU	Events	3-Year Cumulative Incidence	Estimated 3-Year Risk	Risk Difference	Risk Ratio
Initiation cohort intention to treat analysis								
TLD	3711	1.42 (0.83–2.25)	5746	18	0.91% (95% CI .40–1.42)	0.78% (0.38–1.11)	−0.25% (−0.94 to 0.24)	0.75 (0.31–1.30)
TEE	3599	1.92 (0.92–3.00)	6721	28	1.00% (0.61–1.39)	1.03% (0.63–1.55)		
Initiation cohort per-protocol analysis								
TLD	3711	1.33 (0.75–2.17)	5538	16	0.78% (0.30–1.26)	0.66% (0.30–1.18)	−0.33% (−0.92 to 0.38)	0.67 (0.27–1.55)
TEE	3599	1.58 (0.75–2.83)	6014	26	0.97% (0.57–1.36)	1.00% (0.60–1.44)		
Transition cohort intention to treat analysis								
TLD	2837	1.42 (0.83–2.50)	4642	19	1.05 (0.51–1.59)	0.97% (0.52–1.62)	−0.20% (−0.66 to 0.44)	0.83 (0.45–1.39)
TEE	706 615^a^	2.08 (1.17–3.00)	1 391 382^a^	5420	1.19 (1.16–1.23)	1.17% (0.99–1.37)		
Transition cohort per-protocol analysis								
TLD	2837	1.33 (0.75–2.33)	4315	13	0.61% (0.27–0.96)	0.63% (0.29–1.05)	−0.53% (−0.95 to −0.05)	0.54 (0.25–0.96)
TEE	706 615^a^	1.92 (1.00–2.92)	1 308 491	4834	1.13% (1.09–1.16)	1.17% (0.99–1.37)		

^a^Person-trials as individuals could appear in multiple sequential trials while on TEE.

**Table 4.** Baseline Characteristics of Transition Cohort Emulated Target Trial Population and Pseudo-population After Inverse Probability of Treatment Weighting

		Transition Cohort			Sequential Target Trials’ Pseudo-population		
Variable	Level	TEE (n = 706 615)	TLD (n = 2837)	Total	TEE (n = 706 614)	TLD (n = 2753)	Standardized Mean Difference
Age, years	Mean (SD)	44.0 (8.8)	44.4 (8.7)	44.0 (8.8)	44.06 (8.78)	44.80 (8.83)	0.084
Gender	Female (%)	439 607 (62.2)	1643 (57.9)	441 250 (62.2)	439 482.0 (62.2)	1729.76 (62.8)	0.013
Province	Gauteng	332 352 (47.0)	1445 (50.9)	333 797 (47.0)	332 468.9 (47.1)	1300.6 (47.2)	0.024
	Eastern Cape	37 152 (5.3)	171 (6.0)	37 323 (5.3)	37 173.8 (5.3)	156.6 (5.7)	…
	KwaZulu-Natal	184 488 (26.1)	645 (22.7)	185 133 (26.1)	184 386.8 (26.1)	718.8 (26.1)	…
	Mpumalanga	37 540 (5.3)	124 (4.4)	37 664 (5.3)	37 512.4 (5.3)	138.8 (5.0)	…
	Other	67 633 (9.6)	210 (7.4)	67 843 (9.6)	67 570.6 (9.6)	256.4 (9.3)	…
	Western Cape	47 450 (6.7)	242 (8.5)	47 692 (6.7)	47 502.0 (6.7)	182.0 (6.6)	…
Years on ART	Mean (SD)	6.3 (3.6)	7.1 (3.8)	6.3 (3.6)	6.41 (3.57)	7.12 (3.71)	0.194
Scheme benefit level	Network/PMB	213 939 (30.3)	738 (26.0)	214 677 (30.3)	213 827.1 (30.3)	785.0 (28.5)	0.046
	High day to day	65 980 (9.3)	293 (10.3)	66 273 (9.3)	66 004.8 (9.3)	243.3 (8.8)	…
	Low day to day	426 696 (60.4)	1806 (63.7)	428 502 (60.4)	426 782.6 (60.4)	1725.0 (62.7)	…
Initiation VL, copies/mL	0–49	578 225 (81.8)	2418 (85.2)	580 643 (81.8)	580 545.8 (82.2)	2307.1 (83.8)	0.057
	50–999	34 077 (4.8)	180 (6.3)	34 257 (4.8)	34 103.9 (4.8)	114.7 (4.2)	…
	≥1000	0 (0.0)	0 (0.0)	0 (0.0)	465.3 (0.1)	0.0 (0.0)	…
	Missing	94 313 (13.3)	239 (8.4)	94 552 (13.3)	91 499.5 (12.9)	331.5 (12.0)	…
Initiation CD4 count, cells/µL	0–49	126 (0.0)	0 (0.0)	126 (0.0)	137.6 (0.0)	0.0 (0.0)	0.085
	50–199	6959 (1.0)	44 (1.6)	7003 (1.0)	6959.8 (1.0)	13.4 (0.5)	…
	200–349	32 580 (4.6)	148 (5.2)	32 728 (4.6)	32 570.2 (4.6)	117.1 (4.3)	…
	350–499	78 083 (11.1)	311 (11.0)	78 394 (11.0)	78 181.4 (11.1)	280.8 (10.2)	…
	≥500	416 697 (59.0)	1524 (53.7)	418 221 (58.9)	418 994.9 (59.3)	1719.7 (62.5)	…
	Missing	172 170 (24.4)	810 (28.6)	172 980 (24.4)	169 770.4 (24.0)	622.2 (22.6)	…
TB at ART initiation	Yes (%)	205 (0.0)	1 (0.0)	206 (0.0)	201.5 (0.0)	0.0 (0.0)	0.024
Pregnant at ART initiation	Yes (%)	5624 (0.8)	25 (0.9)	5649 (0.8)	5467.7 (0.8)	10.4 (0.4)	0.052
Hypercholesterolemia	Yes (%)	28 259 (4.0)	76 (2.7)	28 335 (4.0)	28 533.4 (4.0)	88.2 (3.2)	0.045
Hypertension	Yes (%)	92 167 (13.0)	288 (10.2)	92 455 (13.0)	92 655.0 (13.1)	362.2 (13.2)	0.001
Diabetes mellitus	Yes (%)	28 930 (4.1)	60 (2.1)	28 990 (4.1)	29 064.3 (4.1)	82.6 (3.0)	0.060
Statin use	Yes (%)	112 691 (15.9)	350 (12.3)	113 041 (15.9)	16 481.0 (2.3)	53.5 (1.9)	0.027
Baseline BMI^a^	Underweight/normal	46 363 (6.6)	285 (10.0)	46 648 (6.6)	47 029.8 (6.7)	218.9 (8.0)	0.102
	Overweight	58 957 (8.3)	352 (12.4)	59 309 (8.4)	59 925.7 (8.5)	270.4 (9.8)	…
	Obese/severely obese	61 580 (8.7)	325 (11.5)	61 905 (8.7)	62 796.5 (8.9)	295.3 (10.7)	…
	Missing	539 715 (76.4)	1875 (66.1)	541 590 (76.3)	536 862.5 (76.0)	1968.6 (71.5)	…

^a^Not included in model to calculate IPTWs in main analysis.

Within the sequential trials, person-trials were followed for a median of 25 (14–36) months, for a total of 1 396 025 person-trial-years. Among those who were allocated to TLD at trial baseline, 302 (10.6%) subsequently changed regimen to an efavirenz-based regimen (n = 202), another dolutegravir-based regimen (n = 65), or another non-dolutegravir-based regimen (n = 35), after a median of 8 (4–13) months. Of those who continued TEE at baseline, 81 326 (11.5%) subsequently changed regimen, to TLD (n = 72 701), another dolutegravir-based regimen (n = 2545), another efavirenz-based regimen (n = 1586), or another non-efavirenz-based regimen (n = 4494), after a median of 14 (7–24) months. There were 19 MACEs with TLD after a median of 9 months (IQR 6.5–15) and 5420 with TEE after a median of 15 months (IQR 7–23). The cumulative incidence of MACE at 3 years with TLD was 1.05 (0.51–1.59) and 1.19 (1.16–1.23) with TEE.

In the sequential trials intention-to-treat analysis, the estimated 3-year risk of MACE was 0.97% (0.52–1.62) with TLD and 1.17% (0.99–1.37) with TEE (RR 0.83 [0.45–1.39]; RD −0.20% [−0.66 to 0.44], Table 3). In the per-protocol analysis, the estimated 3-year risk of MACE was 0.63% (0.29–1.05) with TLD and 1.17% (0.99–1.37) with TEE (RR 0.54 [0.25–0.96]; RD −0.53 [−0.95 to −0.05]). In a sensitivity analysis including baseline BMI (with a category for missing) in the model to calculate IPTWs, there was no meaningful change in results (intention-to-treat analysis RR 0.90 [0.50–1.46]; RD −0.12% [−0.62 to 0.50]). In a sensitivity analysis with an interaction term between gender and treatment allocation in the MACE outcome model, there was no evidence of a difference in the effect of TLD on MACE in women (RR 0.89, 0.36–1.94) and in men (RR 0.76, 0.29–1.27; Supplementary Figure 1B).

## DISCUSSION

In this large South African cohort of PWH, newly initiating and already receiving ART, we found no evidence of an increased risk of MACE over 3 years among people taking TLD compared to TEE.

Our findings align with most previous studies from high-income settings, which have generally not found evidence of increased MACE risk with INSTIs. A systematic review of nine clinical trials with 6647 person-years of follow-up found no evidence of increased serious adverse cardiovascular events with dolutegravir (15/2202, 0.7%) versus other antiretrovirals (8/2215, 0.4%, relative risk 1.69, 95% CI .71–4.03), although numbers were small and study design and comparator antiretrovirals were heterogeneous [23]. Two retrospective cohort studies using North American health insurance data and IPTW among 20 242 [9] and 14 076 [10] new ART initiators found that INSTIs were associated with lower (HR 0.79, 0.64–0.96) [9] or similar (HR 1.30, 0.95–1.88) risk of MACE in the first year or 2 of follow-up. In contrast, a retrospective cohort study of 29 340 individuals in European and Australian cohorts found that INSTI use was associated with an increased risk of MACE in people with 0–6 months (incidence rate ratio [IRR] 1.85, 1.44–2.39) and >12–24 months (IRR 1.46, 1.13–1.68), of cumulative INSTI exposure, before equalizing between 24 and 36 months (IRR 0.89, 0.62–1.29) [4]. While the study design has been questioned [8], these findings have raised concern for global HIV treatment programs and resulted in calls for further studies [24]. Subsequent target trial emulations in North American and European cohorts (n = 87 990 individuals) found similar risks of MACE among people newly initiating INSTI versus non-INSTI ART (4-year aRD 0.01%, −0.43 to 0.36) and among people transitioned to INSTI versus remaining on non-INSTI ART (aRD −0.07%, −0.60 to 0.52) [8], while an emulated target trial using data from 5362 Swiss people found no difference in MACE between those initiating INSTIs versus non-INSTIs (adjusted hazard ratio 0.80, 0.46–1.39) [7].

Our study adds substantially to the evidence base by specifically evaluating the risk of MACE with TLD in an LMIC setting, where this regimen is most widely used. Furthermore, we include people transitioning from TEE to TLD, who make up the majority of people exposed to TLD globally, and directly address the causal question of whether this transition increases MACE risk. Further strengths of our study include the use of comprehensive, longitudinal health insurance claims data that include both primary care treatment and cardiovascular risk factor data, and secondary care and vital statistics data on cardiovascular hospitalizations and deaths. While such health insurance datasets have been used for research in high-income settings, their use in LMICs is more recent [25, 26], providing an opportunity in settings where large research cohorts with long follow-up time have not yet been established. A potential weakness of using health insurance data [25] is use of self-reported medical history for preexisting medical conditions, high levels of missingness for some variables (eg smoking, initiation viral load, BMI), and that our results may not be generalizable to public sector settings, where most people receive HIV care in South Africa [27]. Nevertheless, in private sector settings, stroke diagnosis may be more reliable than in the public sector, where there is often limited access to computed tomography or magnetic resonance imaging scans [28]. We used an emulated target trial methodology to minimize bias in this analysis of observational data. However, we cannot rule out residual confounding, in particular by BMI, which was missing for many participants. Clinicians may have avoided using TLD in people with a high BMI, meaning that the TLD group would have had a lower baseline BMI, which would lower their baseline cardiovascular risk. Furthermore, we did not have complete enough data to estimate baseline predicted cardiovascular risk. However, to calculate the IPTW, we included baseline hypertension, diabetes, and hypercholesterolemia status, which are on the pathway between BMI and cardiovascular risk. While our study is one of the largest in an LMIC to evaluate cardiovascular outcomes among people on ART, with over 6500 individuals receiving TLD, the upper bound of the 95% CI for the risk difference in the transition cohort was 0.44%, meaning we cannot rule out an extra 4.5 MACEs per 1000 individuals with TLD over 3 years. Our cohorts were relatively young, and so larger studies as cohorts age, and with greater follow-up time, are required to determine if TLD associated weight gain translates to higher MACE risk beyond 3 years.

Overall, our results provide reassurance that for the over 20 million people in LMICs who are now using TLD instead of TEE, there is no large increase in MACE risk in the short to medium term. Given the benefits of TLD in terms of viral suppression and current low prevalence of resistance, these findings support the ongoing use of TLD as recommended by WHO.

## Supplementary Material

ciag155_Supplementary_Data

## Supplementary Data

Supplementary materials are available at *Clinical Infectious Diseases* online. Consisting of data provided by the authors to benefit the reader, the posted materials are not copyedited and are the sole responsibility of the authors, so questions or comments should be addressed to the corresponding author.
